# Abscopal effects of thoracic X-ray radiation on spermatogenesis in mice

**DOI:** 10.3389/fphys.2022.984429

**Published:** 2022-08-26

**Authors:** Pan-Pan Lai, Yun-Tao Jing, Ling Guo, Tong-Zhou Qin, Yi-Zhe Xue, Zhao-Wen Zhang, Xing Wang, Xia Miao, Wei Zhang, Gui-Rong Ding

**Affiliations:** ^1^ Department of Radiation Protection Medicine, School of Military Preventive Medicine, Fourth Military Medical University, Xi’an, China; ^2^ Ministry of Education Key Lab of Hazard Assessment and Control in Special Operational Environment, Xi’an, China

**Keywords:** thoracic irradiation, abscopal effect, testicular damage, sperm quality, spermatogenesis

## Abstract

The study aimed to elucidate abscopal effects of thoracic X-ray irradiation on spermatogenesis in mice. Male *C57BL*/*6* mice were randomly divided into sham group and radiation group, and subjected to thorax fractionated X-ray irradiation or sham irradiation with the total dose of 5 Gy/day for each animal for four consecutive days. After irradiation, sperm morphology was observed, and sperm number was counted under microscope, and sperm apoptosis was detected by flow cytometry. Meanwhile, testis index was calculated, testicular morphology was observed using haematoxylin-eosin (HE) staining, and testicular ultrastructure was observed under transmission electron microscopy. The permeability of blood-testis barrier (BTB) was detected by Evans Blue fluorescence colorimetry. The protein levels of Bcl-2 associated X protein (Bax), B-cell leukemia-lymphoma-2 (Bcl-2) and Cleaved caspase 3, promyelocytic leukaemia zinc finger (PLZF) and c-kit proto-oncogene (c-kit) in testes were determined by western blotting (WB). The location of apoptotic cells was confirmed by terminal deoxynucleotidyl transferase (TdT) enzymaticated dUTP nick end labelling (TUNEL) assay. The levels of tumor necrosis factor alpha (TNF-α), transforming growth factor-β1 (TGF-β1), interleukin 10 (IL-10) were measured by enzyme-linked immunosorbent assay (ELISA). The levels of Total superoxide dismutase (T-SOD) and malondialdehyde (MDA) were measured by the biochemical assay kit. Compared with sham group, the sperm quality of mice in radiation group showed decreased number and survival rate, along with increased abnormality and total apoptosis rate. The testis index of irradiated mice was lower, the testicular apoptosis was increased, and their testicular histology and ultrastructure was severely damaged. The permeability of BTB was increased, the level of PLZF in testis was decreased, and the level of c-kit was increased by irradiation. After irradiation, the levels of TNF-α, TGF-β1, IL-10, T-SOD and MDA in testes were significantly changed. Taken together, abscopal effects of thoracic X-ray irradiation on spermatogenesis were obvious, which could decrease sperm quality and damage testicular morphology and increase the permeability of BTB, and a series of inflammation and oxidative stress factors were involved in the process. These findings provide novel insights into prevention and treatment for male reproductive damage induced by clinical thoracic irradiation.

## 1 Introduction

Lung cancer as a kind of malignant tumor is still top-ranked morbidity and mortality in cancers worldwide and it is worth noting that the number of young patients with lung cancer gradually increases (Dee et al., 2022). Radiation therapy is one of the main treatment modalities for lung cancer and approximately 50% of lung cancer patients require thoracic radiation therapy (Begg et al., 2011). In addition, patients with other thoracic tumors like esophagus cancer, thymoma, or mediastinal lymphoma also require thoracic radiation therapy (Abuodeh et al., 2016).

At present, thoracic radiation therapy modes applied in clinical practice mainly include conventional fractionation and hypofractionation, and the single irradiation dose of them is 2 Gy and more than 2.5 Gy respectively. However, conventional fractionated radiotherapy is restricted by its long course and high cost for patients. Therefore, hypofractionated radiotherapy has been promoted in radiotherapy of thoracic tumors with advantage of the increased ratio of radiotherapy and low cost (Vischioni et al., 2019; Wei et al., 2020). Hypofractionated radiotherapy can not only destroy the tumor in the irradiated area, but also may cause damage to remote unirradiated areas, known as abscopal effects of ionizing radiation (Yilmaz et al., 2019), such as the regression of the metastatic tumors and the damage of normal tissue in the unirradiated area (Liu et al., 2018; Ashrafizadeh et al., 2020). In terms of mechanism, it is reported that TGF-β was involved in the testicular injury induced by thoracic X-ray fractional irradiation in rats (Zhang et al., 2019b), and other studies revealed that inflammatory factors, oxidative stress and exosomes may also play important roles in abscopal effects induced by ionizing radiation (Najafi et al., 2016; Sisakht et al., 2020).

It is reported that the testis is extremely sensitive to ionizing radiation, even low-dose direct radiation can also disturb spermatogenesis, and high-dose direct radiation can severely damage spermatogenesis, resulting in sperm apoptosis, oligospermia and even azoospermia (Marzban et al., 2017). However, the damage of the male reproductive system induced by thoracic ionizing radiation and its underlying mechanism has not been fully elucidated so far. To bridge the gap, we conducted the study, hoping to provide novel insights into prevention and treatment of male reproductive injury induced by clinical radiotherapy.

## 2 Materials and methods

### 2.1 Animals

Healthy adult male C57BL/6 mice aged 6–8 weeks (certificate number: SYXK-2019-001) were purchased from the laboratory animal center of Fourth Military Medical University (Xi’an, China). Mice were housed (four mice per cage) in the animal facility and maintained at 25°C with 12 h light-dark cycling. Mice were randomly divided into sham group and radiation group, 12 mice in each group. All animal experiments were censored and approved by the Animal Welfare and Ethics Committee of Fourth Military Medical University (Xi’an, China).

### 2.2 The radiation protocol

The thorax of mice was irradiated by the X-ray irradiator (RAD Source RS 2000 series, Suwanee, United States) with working electric current 25 mA and working voltage 160 kV. Mice in radiation group was subjected thoracic X-ray irradiation consecutively at 5 Gy/d and a dose rate of 2.33 Gy/min for 4 d, which was monitored in real time by a radiation dosimeter (Radcal Accu-Dose, United States). Meanwhile, mice in sham group were under the same experimental condition without irradiation.

### 2.3 The sample collection and testis index calculation

The body weight of each mouse was recorded every 3 days. All mice were euthanized with 1% sodium pentobarbital (50 mg/kg) at 1 W, 3 and 5 W after thoracic X-ray irradiation. The bilateral testes were rapidly separated from the surrounding connective tissue and excised after whole-body perfusion with 0.9% sodium chloride. Testicular tissues were rinsed with pre-chilled phosphate-buffered saline (PBS), weighed using the balance, snap-frozen in liquid nitrogen, and stored at −80°C until analysis. The testis index was calculated by “bilateral testis weight (g) divided by body weight (g)” and express as percentage.

### 2.4 Haematoxylin-eosin staining

After anesthesia, the heart was perfused with 0.9% NaCl solution, and then slowly perfused with 4% paraformaldehyde in the same position. Bilateral testes were fixed in Bouin’s solution (Lilai, Chengdu, China) for 24 h, then the fixed testes were routinely dehydrated, embedded in paraffin and then serially sectioned on a rotary microtome (RM2135, Leica, Heidelberg, Germany) at a thickness of 4 μm. Subsequently tissue sections were deparaffinized at 65°C for 2 h, rehydrated in graded ethanol, and stained with hematoxylin-eosin (HE) according to routine protocols. Finally, histological changes were observed by the inverted microscopy (Leica).

### 2.5 Histological analysis

After HE staining, the diameter of seminiferous tubules and the height of seminiferous epithelium were randomly selected from 50 round or nearly round seminiferous tubules using ImageJ 1.43 u software (NIH, MD, United States). The diameter was calculated as the mean of the major and minor axes of the seminiferous tubules, and the height of the seminiferous epithelium was calculated by (average diameter—average inner diameter) of seminiferous tubule/2.

### 2.6 Observation of testicular ultrastructure by transmission electron microscopy

After intraperitoneal anesthesia, testes of mice were separated and trimmed to 2 mm × 2 mm × 2 mm samples, pre-fixed with 3% glutaraldehyde and 1% osmium acid, dehydrated in a graded series of acetone (30, 50, 70, 80, 90, 95, 100%) and then embedded in Araldite. Ultrathin slices (60 nm thick) were stained for 20 min at room temperature. A transmission electron microscope (JEM-1400FLASH; JEOL Ltd., Tokyo, Japan) was used to observe the ultrastructure of seminiferous tubules.

### 2.7 TUNEL assay

The apoptotic cells were stained with TUNEL assay using an *in situ* Cell Death Detection Kit (Roche, Basel, Switzerland). The paraffin sections of mice testes were dewaxed and rehydrated for antigen retrieval with proteinase K working solution, the sections were permeabilized by 0.3% Triton X-100 (ST795, Beyotime, Shanghai, China), and subsequently treated with 40 μl TUNEL reaction mixture for 60 min at 37°C. The images in the testicular tissue were captured with the fluorescence microscope (Leica), and the average fluorescence intensity was calculated using the ImageJ software.

### 2.8 Western blotting

Total testicular protein (*n* = 4 for each group) was extracted and quantified. Equal amounts of testicular samples (30 μg) were subjected to 10–12% Bis-Tris gel electrophoresis and transferred to polyvinylidene fluoride immunoblot membranes. The membranes were blocked with 5% non-fat milk solution for 2 h at room temperature and treated with primary antibodies overnight at 4°C. Primary antibodies including β-actin (1:1000, Cell Signaling Technology, United States), Bcl-2 (1:2000, Proteintech, Wuhan, China), Bax (1:4000, Proteintech, Wuhan, China), and PLZF (1:2000, Proteintech, Wuhan, China), c-kit (1:1000, Affinity, United States) and Cleaved Caspase-3 (1:1000, Affinity, United States), remove excess primary antibodies and subsequently treated with appropriated second antibody (1:5000, CWBIO, China) for 2 h at room temperature, and then visualized using an enhanced chemiluminescence detection kit. The results were analyzed by the Quantity One 4.62 software (Bio-Rad, CA, United States).

### 2.9 Evaluations of sperm quality

At different time points after thoracic X-ray irradiation, the bilateral epididymal cauda of each mouse were isolated, gently cut, collected in 1 ml of sperm culture solution (Millipore, MA, United States), and then incubated at 37°C for 40 min. The sperm suspension was pipetted and filtered through 38 μm pore diameter to remove tissue fragments. A solution containing the sperm was then flushed dropped into a disposable counting plate, and the sperm number was manually monitored and counted under the inverted microscope (Leica). Abnormal sperm morphology was measured by taking the sperm suspension smear, fixed, stained, and then counted under the inverted microscope (Leica). The types of abnormal sperm morphology observed in this study mainly included the folded-tail, hookless, amorphous and double-head phenotypes. In addition, a FITC annexin V apoptosis detection kit Ι (BD Pharmingen, CA, United States) was applied to quantify the survival rate and apoptosis rate of sperm according to the manufacture’s instruction.

### 2.10 Immunohistochemistry

After being paraffinized and rehydrated and quenched, testicular sections were processed by antigen retrieval using citrate buffer in a high-power microwave oven, treated with 3% bovine serum albumin at room temperature for 1 h, and incubated with the primary antibody of c-kit (1:1000, Affinity) at 4°C overnight, and subsequently treated with corresponding second antibody (1:5000, CWBIO, China). Then the tissue sections were stained by diaminobenzidine, hematoxylin, and observed under the inverted microscope (Leica), and positive cell percentage of c-kit was counted by ImageJ software.

### 2.11 Evans blue fluorescence

Two% Evans Blue (EB) solution (Sigma-Aldrich, MO, United States) was injected into the tail vein at 4 ml/kg and circulated in the body for 30 min. After the perfusion completion, bilateral testes were dissected out, put into freezing medium, stored at −80°C in the dark, and then subsequently serially sectioned on a rotary microtome (RM2135, Leica, Heidelberg, Germany) at a thickness of 15 μm. EB exudation in seminiferous tubules of testis was observed under the fluorescence microscope (Leica).

### 2.12 Detection of inflammatory factor by ELISA

Inflammatory factor levels in lung, serum and testis were detected. Lung and testis tissue (approximately 100 mg; *n* = 6 per group) were homogenized in a homogenizer under precooled conditions to extract total proteins. Blood samples of mice were centrifuged at 3000 rpm for 15 min at 4 °C to obtain serum (*n* = 9 per group). After that, the levels of TNF-α, TGF-β1 and IL-10 in lung, serum, and testis were measured with the ELISA kits (Elabscience, Wuhan, China) according to the manufacturer’s instructions.

### 2.13 Detection of oxidative stress levels by assay kit

Oxidative stress levels in lung, serum and testis were detected. The Sample collection of lung (*n* = 6 per group), serum (*n* = 9 per group) and testis (*n* = 6 per group) is the same as ELISA. After that, the levels of T-SOD and MDA in lung, serum, and testis were measured with the Assay kits (Elabscience, Wuhan, China) according to the manufacturer’s instructions.

### 2.14 Statistical analysis

All measurement data are expressed as the mean and standard deviation (mean ± SD) and were analyzed with the SPSS 20.0 statistical software (SPSS Inc., Chicago, IL, United States). All graphs were generated using the GraphPad Prism 9.0 software (San Diego, CA, United States), and the results were considered statistically significant when *p* < 0.05. For statistical analysis, the weight of mice was monitored by two-way ANOVA with repeated measures, the Western Blot results were compared between two groups by a one-sample t test, and the other results were compared between two groups by a two-tailed student’s t-test. All subjective analyses were performed by individuals blinded to the experimental groups.

## 3 Results

### 3.1 Testicular histology damage induced by thoracic X-ray irradiation

**FIGURE 1:**
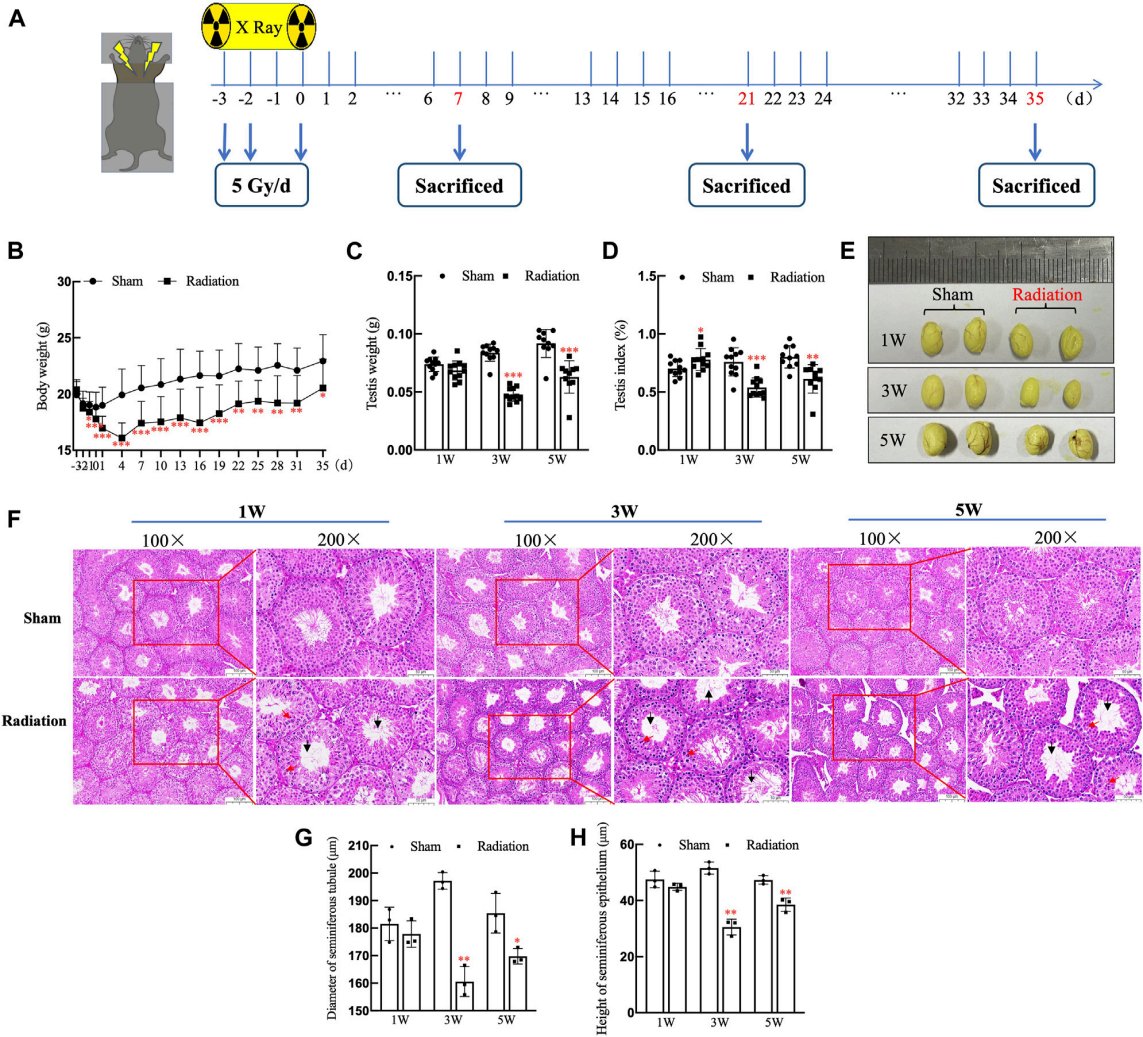
Abscopal effects of thoracic X-ray irradiation on testicular histology in mice. **(A)** Time schedule of irradiation for mice. **(B)** Body weight of mice at different time points, *n* = 10 for each group. **(C)** Testis weight, *n* = 10 for each group. **(D)** Testis index, *n* = 10 for each group. **(E)** Testis volume, *n* = 3 for each group. **(F)** HE staining of testes, *n* = 3 for each group, bar = 100 μm (×100); 50 μm (×200). **(G–H)** Diameter of seminiferous tubules and height of seminiferous epithelium calculated randomly from 50 round or nearly round cross-sections of the seminiferous tubules (long axis: short axis <1.2:1) for each group. The values are expressed as the mean ± SD, *p < 0.01; **p < 0.01; ***p < 0.001 vs. sham group.

The time schedule of thoracic X-ray irradiation and operations on mice (Figure 1A). During the whole experiment, the body weights of the mice on the 3rd day significantly decreased, and it decreased to the lowest level on the 4th day, subsequently it began to increase slowly, but until the 35th day, the body weight of mice in radiation group was still significantly lower than the sham group (Figure 1B; *p* < 0.05). The testis volume, testis weight and testis index were significantly decreased at 3 and 5 W after thoracic X-ray irradiation (Figures 1C–E; *p* < 0.05 or 0.001). At 1 W time point, since the body weights of the mice in the radiation group significantly decreased, while testis weight did not change, the testis index of radiation group showed higher than the sham group. The results of HE staining showed that at different time points after thoracic X-ray irradiation, the testicular structure of the mice in sham group was normal, the spermatogenic cells at all levels were regularly arranged, and there were a large number of mature spermatozoa in the seminiferous tubule lumen, while there were obvious pathological changes in radiation group, such as vacuolization of seminiferous tubules, disordered arrangement of spermatogenic cells at all levels (Figure 1F). In addition, the seminiferous tubule diameter and seminiferous epithelium significantly reduced (Figures 1G,H; *p* < 0.05 or 0.01) after thoracic X-ray irradiation. The above results indicate that the structure of testis damaged after thoracic X-ray irradiation.

### 3.2 Testicular ultrastructure damage induced by thoracic X-ray irradiation

**FIGURE 2:**
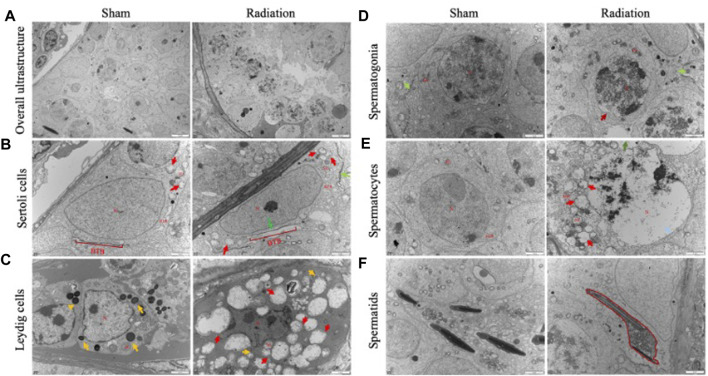
Abscopal effects of thoracic X-ray irradiation on testicular ultrastructure in mice. **(A)** Overall ultrastructure of seminiferous tubules. Bar = 10 μm. **(B–F)** Ultrastructure of Sertoli cells, Leydig cells, spermatogonia, spermatocytes and spermatids. Bar = 2 μm, *n* = 3 for each group. N, nucleus; Mi, mitochondrion; RER, rough endoplasmic reticulum, BTB, blood-testis barrier. Lipid droplets↑, Structurally disrupted blood-testis barrier↑, mitochondrial swelling↑, chromatin dissolution↑.

The results of transmission electron microscopy of testicular tissue showed that the overall ultrastructure of seminiferous tubules in testis of sham group was normal and intact. For radiation group, the overall ultrastructure of the seminiferous tubules was severely damaged, the gaps appeared between the spermatogenic cells (Figure 2A), The structure of BTB was incomplete (Figure 2B); lipid droplets were found in Leydig cells (Figure 2C); the mitochondria were significantly swollen, the chromatin were dissolved, the spermatocytes were necrotic (Figures 2D,E); the sperm cells were destroyed with the cytoplasmic content was lost (Figure 2F). It is suggested that thoracic X-ray irradiation can damage the ultrastructure of testis in mice.

### 3.3 Testicular cell apoptosis induced by thoracic X-ray irradiation

**FIGURE 3:**
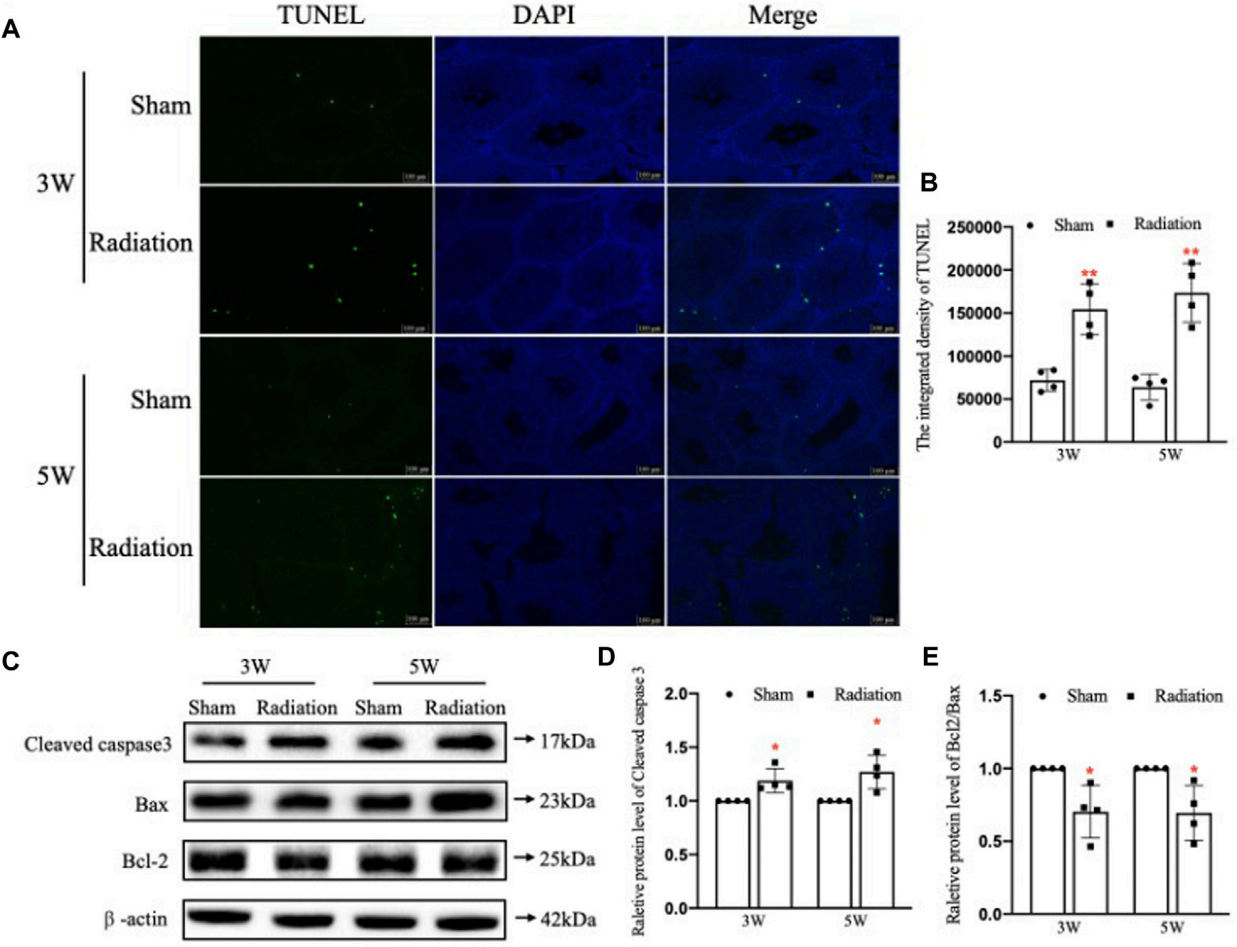
Abscopal effects of thoracic X-ray irradiation on testicular cell apoptosis in mice. **(A,B)** TUNEL staining and average fluorescence intensity, Bar = 100 μm. **(C–E)** Relative protein levels of Bcl-2, Bax, Bcl-2/Bax and Cleaved caspase three detected by WB, *n* = 4 for each group. The values are expressed as the mean ± SD, *p < 0.01; **p < 0.01; ***p < 0.001 vs. sham group.

The results of TUNEL staining showed that the number of apoptotic testicular cells increased at 3 and 5 W after thoracic X-ray irradiation in radiation group compared with sham group, and the apoptotic testicular cells were located at the outermost seminiferous tubules (Figures 3A,B; *p* < 0.01). In addition, WB results (Figure 3C) showed that the protein level of Cleaved Caspase-3 was significantly up-regulated (Figure 3D; *p* < 0.05) and the Bcl-2/Bax ratio significantly decreased (Figure 3E; *p* < 0.05) at 3 and 5 W after thoracic X-ray irradiation compared with sham group, which were consistent with TUNEL results. These results suggest that thoracic X-ray irradiation can increase testicular cell apoptosis.

### 3.4 Sperm quality decrease induced by thoracic X-ray irradiation

**FIGURE 4:**
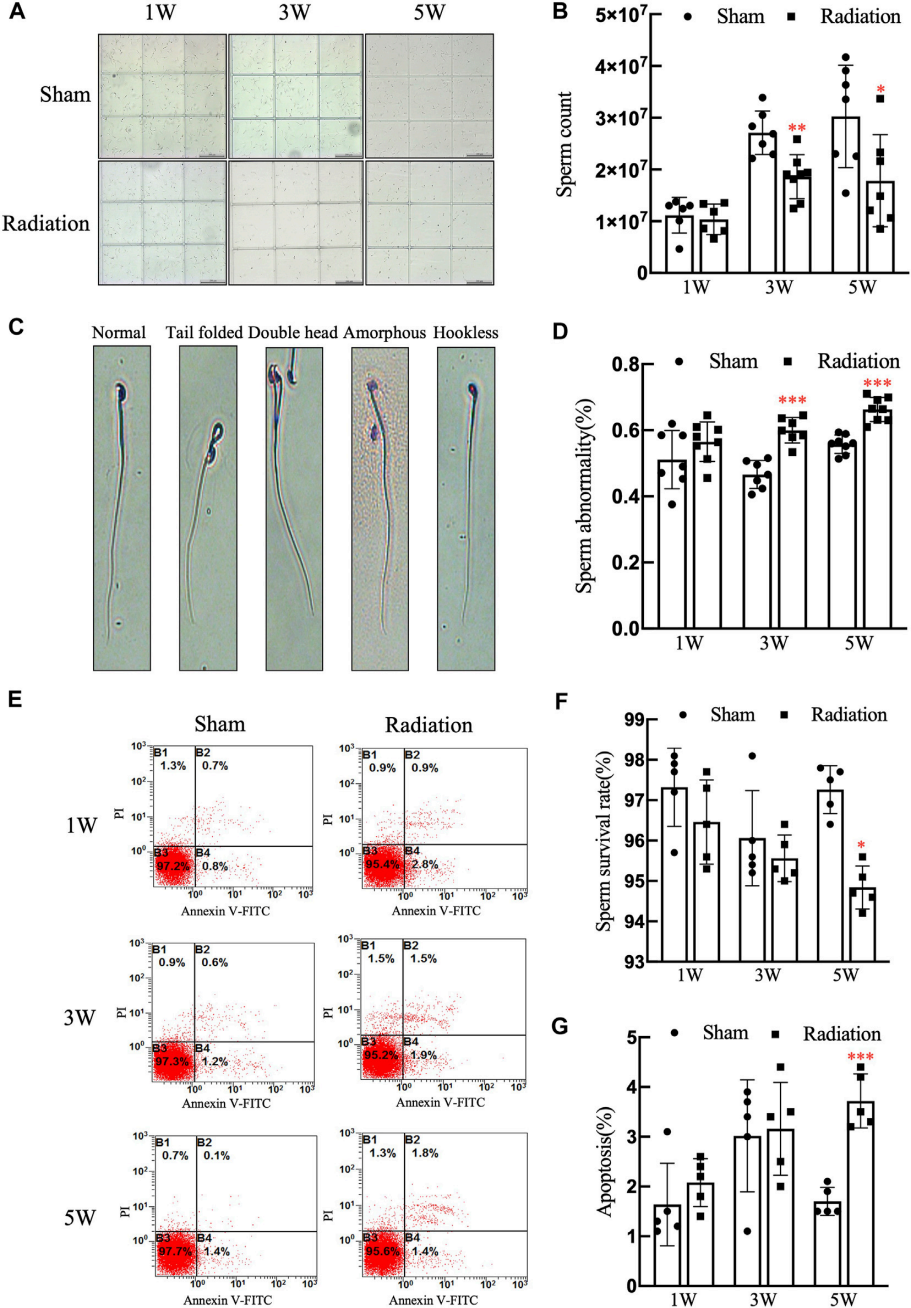
Abscopal effects of thoracic X-ray irradiation on sperm quality in mice **(A)** Representative pictures of sperm count, Bar = 200 μm (×100). **(B)** Analysis of sperm count. *n* = 7 for each group. **(C)** Typical types of abnormal sperm morphology, including the folded-tail, hookless, amorphous, double-head and double-tail phenotypes. **(D)** Sperm abnormality, *n* = 7 for each group. **(E)** Representative pictures of sperm apoptosis detected by FCM. **(F,G)** Survival rate, apoptosis rate of sperm, *n* = 5 for each group. The values are expressed as the mean ± SD, *p < 0.01; **p < 0.01; ***p < 0.001 vs. sham group.

The statistical results of sperm quality of mice showed that compared with sham group, there was no obvious difference in sperm count at 1 W, while it significantly decreased at 3 and 5 W (Figures 4A,B; *p* < 0.001) in radiation group; typical types of abnormal sperm morphology observed in this study are shown in Figure 4C, the sperm abnormality rate was significantly increased at 3 and 5 W (Figure 4D; *p* < 0.05 or 0.01); the results of flow cytometry showed that the sperm survival rate of mice significantly decreased at 5 W (Figure 4F; *p* < 0.05), and the apoptosis rate was increased significantly at 5 W after thoracic X-ray irradiation (Figure 4G; *p* < 0.001). It is suggested that thoracic X-ray irradiation can decrease sperm quality in mice.

### 3.5 Spermatogonia stem cells damage induced by thoracic X-ray irradiation

**FIGURE 5:**
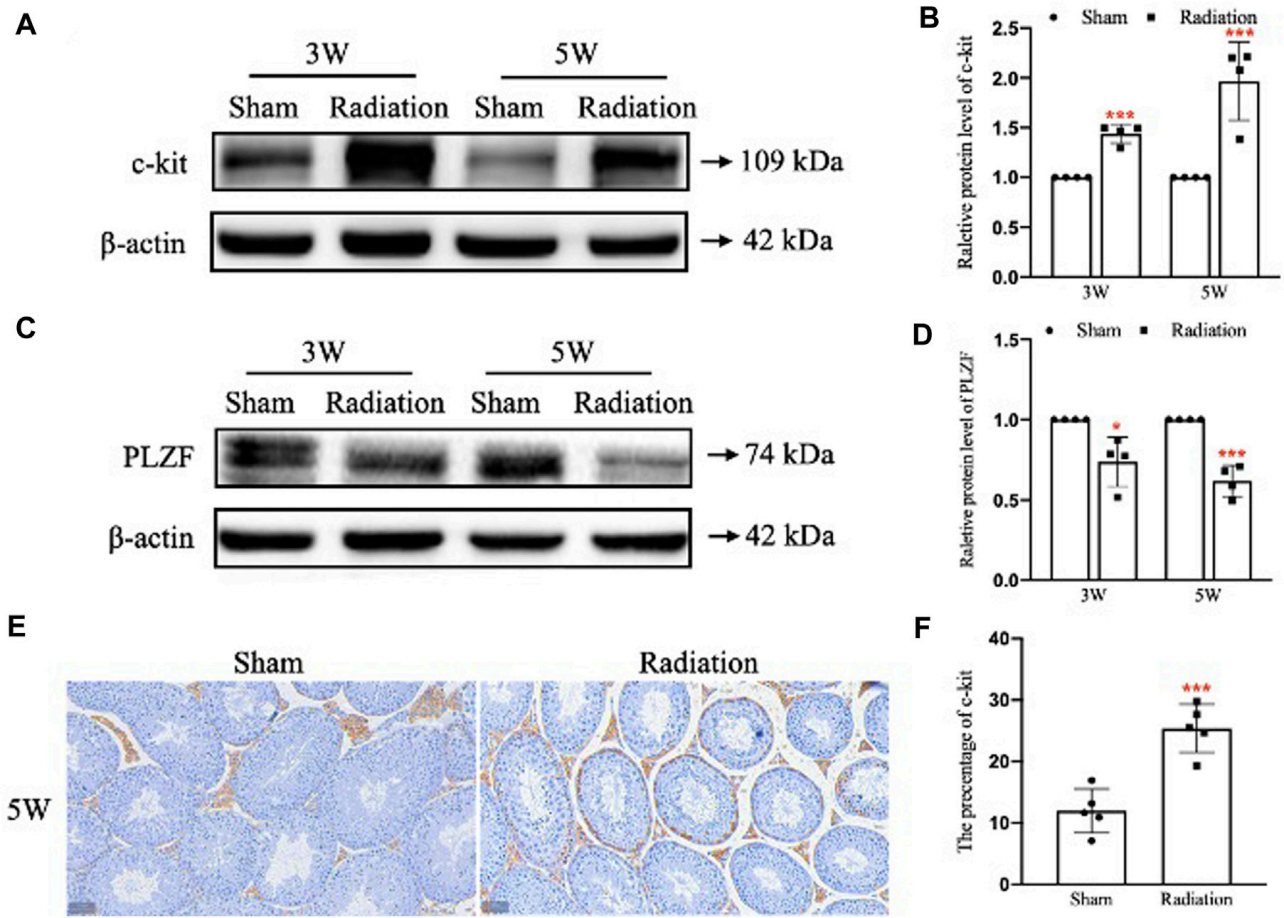
Abscopal effects of thoracic X-ray irradiation on spermatogonia stem cells in mice. **(A,B)** Relative protein level of c-kit detected by WB, *n* = 4 for each group. **(C,D)** Relative protein level of PLZF detected by WB, *n* = 4 for each group. **(E,F)** Immunofluorescence of c-kit and average fluorescence intensity, Bar = 50 μm (×200). The values are expressed as the mean ± SD, *p < 0.01; **p < 0.01; ***p < 0.001 vs. sham group.

WB results showed that compared with sham group, the protein level of c-kit was significantly up-regulated (Figures 5A,B; *p* < 0.001); the protein level of PLZF was significantly down-regulated at 3 and 5 W after thoracic X-ray irradiation (Figures 5E,F; *p* < 0.05 or 0.001). In addition, immunohistochemical results showed that the percentage of positive cells for c-kit protein increased at 5 W after thoracic X-ray irradiation (Figures 5C,D; *p* < 0.001), which was consistent with the results of WB. The results above suggest that thoracic X-ray irradiation can damage the proliferation and differentiation of spermatogonia stem cells.

### 3.6 The permeability of blood-testis barrier alteration induced by thoracic X-ray irradiation

**FIGURE 6:**
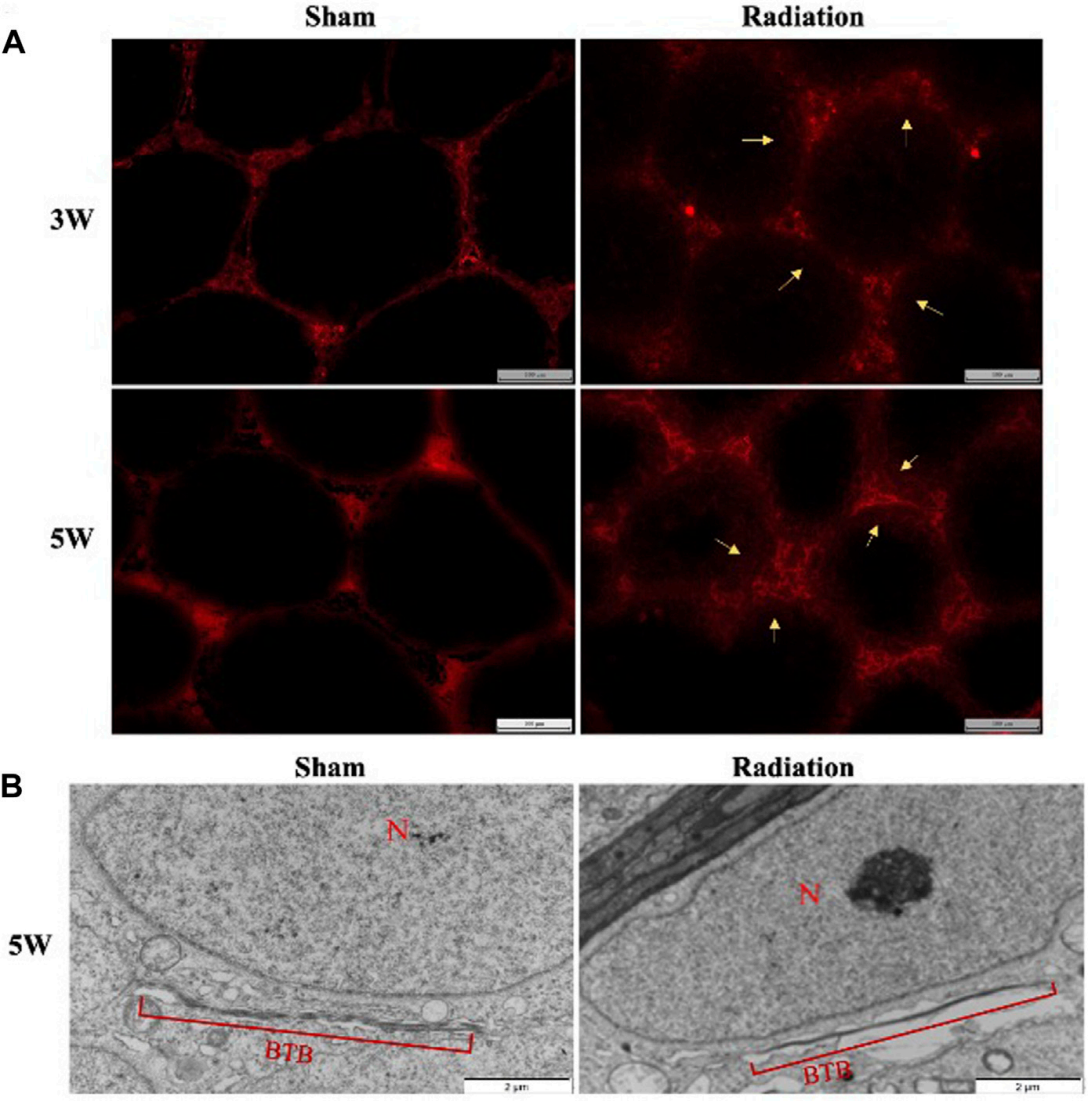
Abscopal effects of thoracic X-ray irradiation on the permeability of BTB in mice. **(A)** Detection of BTB permeability by EB fluorescence chromogenic assay, Bar = 100 μm (200 ×), *n* = 4 for each group. **(B)** TEM observation of BTB, Bar = 100 μm, N: nucleus; BTB: blood-testis barrier, *n* = 3 for each group. The values are expressed as the mean ± SD, *p < 0.01; **p < 0.01; ***p < 0.001 vs. sham group.

There was no obvious EB exudation in the seminiferous tubule in sham group at 3 and 5 W after thoracic X-ray irradiation; a large number of EB showed diffuse exudation in radiation group (Figure 6A). The results of transmission electron microscopy of testes showed that the structure of BTB was obviously destroyed (Figure 6B). It is suggested that thoracic X-ray irradiation can increase the permeability of BTB in testis of mice.

### 3.7 Inflammatory factors alteration induced by thoracic X-ray irradiation in lung, serum and testis

**FIGURE 7:**
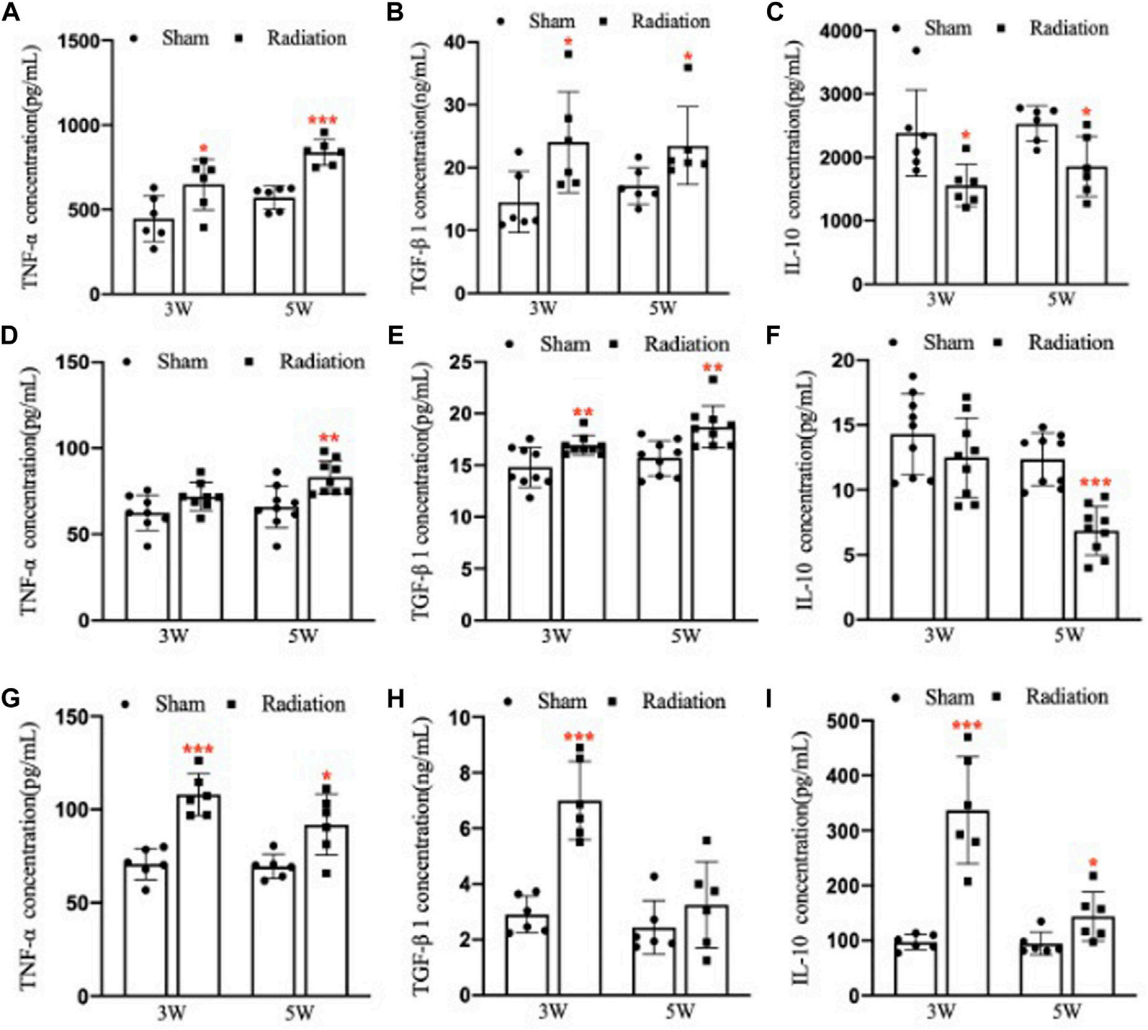
Abscopal effects of thoracic X-ray irradiation on the inflammatory factors in mice **(A)** The concentration of TNF-α in the lung of mice, *n* = 6 for each group. **(B)** The concentration of TGF-β1 in the lung of mice, *n* = 6 for each group. **(C)** The concentration of IL-10 in the lung of mice, *n* = 6 for each group. **(D)** The concentration of TNF-α in the serum of mice, *n* = 9 for each group. **(E)** The concentration of TGF-β1 in the serum of mice, *n* = 9 for each group. **(F)** The concentration of IL-10 in the serum of mice, *n* = 9 for each group. **(G)** The concentration of TNF-α in the testis of mice, *n* = 6 for each group. **(H)**The concentration of TGF-β1 in the testis of mice, *n* = 6 for each group. **(I)** The concentration of IL-10 in the testis of mice, *n* = 6 for each group. The values are expressed as the mean ± SD, *p < 0.01; **p < 0.01; ***p < 0.001 vs. sham group.

Compared with sham group, the concentration of TNF-α and TGF-β1 in lung significantly increased (Figures 7A,B; *p* < 0.05 or 0.001), and the concentration of IL-10 significantly decreased (Figure 7C; *p* < 0.05) at 3 and 5 W after thoracic X-ray irradiation. The concentration of TNF-α in serum of mice significantly increased at 5 W (Figure 7D; *p* < 0.01), the concentration of TGF-β1 significantly increased at 3 and 5 W (Figure 7E; *p* < 0.01), and the concentration of IL-10 significantly decreased at 5 W after thoracic X-ray irradiation (Figure 7F; *p* < 0.001), The concentration of TNF-α, TGF-β1 and IL-10 in testes of mice significantly increased at 3 and 5 W after thoracic X-ray irradiation (Figures 7G–I; *p* < 0.05 or 0.001). All results suggested that after thoracic X-ray irradiation could cause serious inflammatory response in lung, serum and testis of mice.

### 3.8 Oxidative stress levels alteration induced by thoracic X-ray irradiation in lung, serum and testis

**FIGURE 8:**
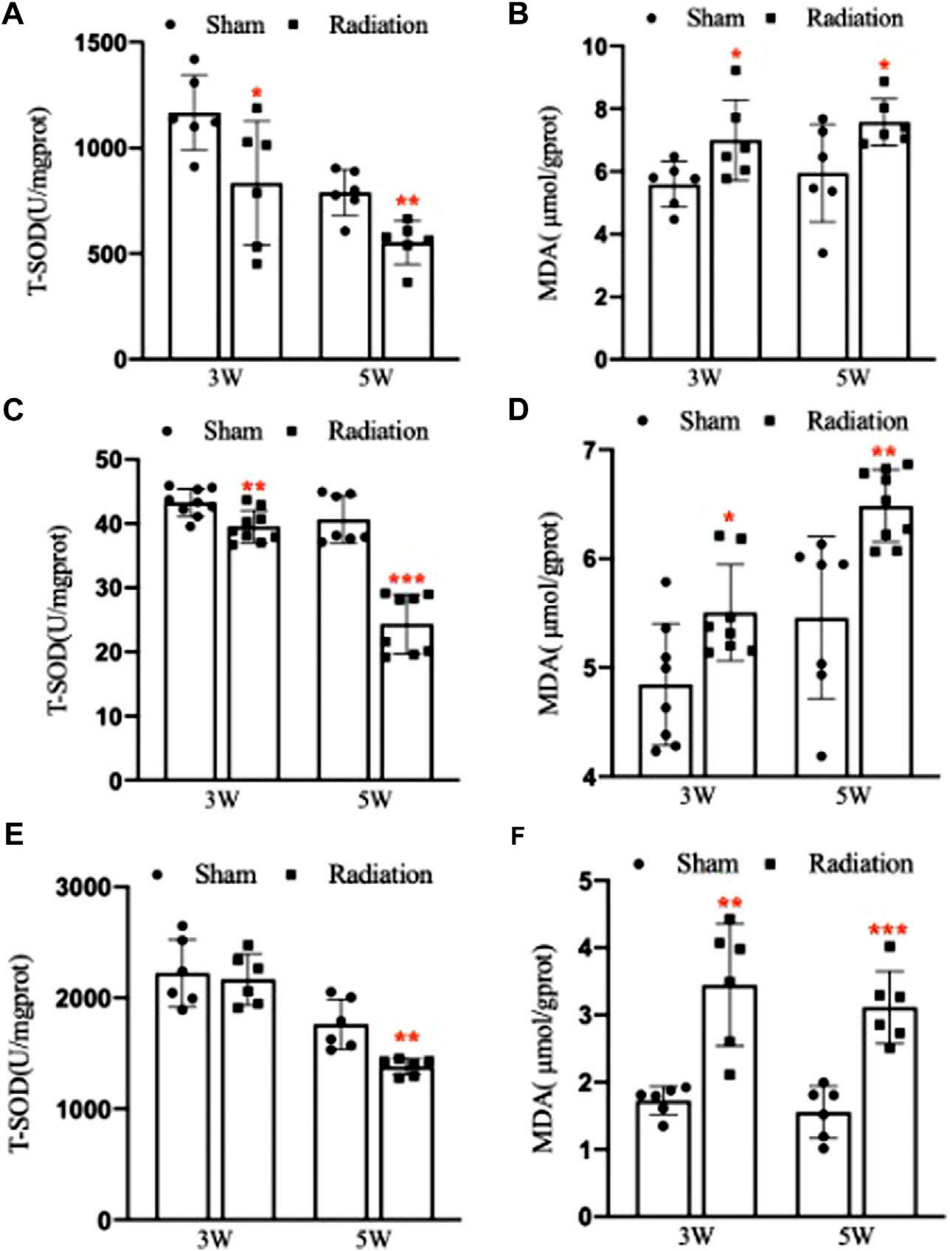
Abscopal effects of thoracic X-ray irradiation on oxidative stress levels in mice. **(A)** The concentration of T-SOD in lung of mice, *n* = 6 for each group. **(B)** The concentration of MDA in lung of mice, *n* = 6 for each group. **(C)** The concentration of T-SOD in serum of mice, *n* = 9 for each group. **(D)** The concentration of MDA in serum of mice, *n* = 9 for each group. **(E)** The concentration of T-SOD in testis of mice, *n* = 6 for each group. **(F)** The concentration of MDA in testis of mice, *n* = 6 for each group. The values are expressed as the mean ± SD, *p < 0.01; **p < 0.01; ***p < 0.001 vs. sham group.

Compared with sham group, the concentration of T-SOD in lung significantly decreased (Figure 8A; *p* < 0.05 or 0.01), and the concentration of MDA significantly increased at 3 and 5 W after thoracic X-ray irradiation (Figure 8B; *p* < 0.05). The concentration of T-SOD in serum significantly decreased (Figure 8C; *p* < 0.01 or 0.001), and the concentration of MDA significantly increased at 3 and 5 W after thoracic X-ray irradiation (Figure 8D; *p* < 0.05 or 0.01). The concentration of T-SOD in testis significantly decreased at 5 W (Figure 8E; *p* < 0.01), and the concentration of MDA significantly increased at 3 and 5 W after thoracic X-ray irradiation (Figure 8F; *p* < 0.01 or 0.001). All results suggested that thoracic X-ray irradiation could alter the levels of oxidative stress in lung, serum and testis of mice.

## 4 Discussion

The concept of abscopal effects was first put forward by Mole team in 1953 when irradiation effects was observed in unirradiated areas (Mole, 1953), unexpectedly causing damage to metastatic tumors or abscopal normal tissues (Mohye El-Din et al., 2017; Chuang et al., 2018; Yin et al., 2020). Thereby it is a big concern on the unclarified spermatogenesis damage induced by thoracic X-ray irradiation especially for young patients. Our study found that thoracic X-ray irradiation could damage testicular structure and function in mice, and finally lead to spermatogenesis deficiency.

Spermatogenesis originated from the testicular seminiferous epithelium that is mainly composed of Sertoli cells and spermatogenic cells. We found that after thoracic X-ray irradiation, the body weight and testis weight and testis index of mice significantly declined, and the structure and function of their testes was obviously damaged. The damage of mice testes worsened at 3 and 5 W after thoracic X-ray irradiation. Song’s team also found that right thorax fractionated X-ray irradiation could damage the testicular structure at different time points, but the peak emerged at 7 d after thoracic X-ray irradiation, and subsequently the injury begun to recover gradually (Song et al., 2021). Inconsistent timing of damage of the testis with this study, it is considered that the time of ionizing radiation-induced abscopal effects on testis damage is inconsistent due to the different thoracic irradiation area and irradiation dose fraction mode. In a previous study, fractionated X-ray cranial irradiation (5 Gy × 4) in mice was found to damage the structure and function of the testis, ultimately reducing sperm quality in mice at 4 W (Guo et al., 2021). This is consistent with our results, suggesting that the testis is the abscopal target of thoracic ionizing radiation. In order to further study the abscopal effects of thoracic X-ray irradiation on testicular tissue, we observed the ultrastructure of testes and found that the overall structure of testicular seminiferous tubules was damaged, including the destruction of the blood-testis barrier and abnormal sperms, suggesting thoracic X-ray irradiation could damage testicular ultrastructure.

Apoptosis is a process of programmed cell death and regulated by genes related with normal development of the body, which plays an important role in individual survival and homeostasis. Ionizing radiation can induce apoptosis in a variety of normal tissues and organs, but its effects on germ cells apoptosis is unusually significant (Liu et al., 2007). It was reported that after right thoracic X-ray irradiation in rats, the protein levels of caspase-3 and caspase-8 in rat testes were increased, and the apoptosis of TM4 cells was also significantly increased after culturing using irradiated rat serum *in vitro*, indicating that thoracic ionizing radiation could induce abscopal apoptosis of testicular tissue cells (Zhang et al., 2019b). Our study also showed that the apoptosis of testicular seminiferous epithelial cells was significantly increased at 3 and 5 W after thoracic X-ray irradiation in mice, along with a significant increase in the protein level of Cleaved caspase-3, and the apoptosis of spermatogenic cells is located around the seminiferous tubules, further elucidating abscopal effects of thoracic X-ray irradiation on the apoptosis of spermatogenic cells.

The main function of the testis is maintaining spermatogenesis, in which germ-line cells and Sertoli cells at different stages are essential for normal spermatogenesis (Nishimura and L'Hernault, 2017). The total number, abnormality rate, and survival rate and of sperms are main indicators for evaluating sperm quality. Tamminga et al. found that X-ray irradiation on rat hippocampus could affect sperm quality (Tamminga et al., 2008). The results of this study showed that the sperm count significantly decreased, the abnormality rate and apoptosis rate of sperm increased in mice at 3 and 5 W after thoracic X-ray irradiation, but the survival rate of sperms was still greater than 95%, indicating that thoracic ionizing radiation increased the apoptosis rate of sperm through intervening with spermatogenesis process other than directly damaging mature sperms. It was reported that the sperm count decreased, the abnormality rate of sperm increased at 5 d after irradiation of ^12^C^6+^ ions or ^60^Co γ-rays radiation on the brain of mice, which is consistent with our results (Zhang et al., 2006).

Spermatogonial stem cells (SSCs) are located in the basement membrane of testicular seminiferous tubules. They have the ability to self-replicate to maintain renewal and to differentiate into spermatocytes. PLZF is essential for self-renewal of SSCs (Song et al., 2020), and C-kit plays vital role in differentiation of SSCs (Raucci and Di Fiore, 2007). Therefore, both PLZF and C-kit were used to evaluate the damage of SSCs in this study. The results showed that the expression of PLZF in testes was significantly decreased at 3 and 5 W after thoracic X-ray irradiation, suggesting that the proliferation of SSCs was inhibited. In addition, the expression of c-kit in testes significantly increased at 3 and 5 W after thoracic X-ray irradiation, indicating that the differentiation of SSCs was promoted. The above data suggested that the abscopal effects of thoracic X-ray irradiation could affect the proliferation and differentiation of SSCs.

BTB located between seminiferous tubules and capillaries is a kind of vital blood barrier structure for supporting spermatogenesis, with important functions in controlling biomolecules entry and ensuring stable microenvironment for spermatogenesis (Chen et al., 2018; Li et al., 2018). Our study indicated that thoracic X-ray irradiation could change the permeability of BTB and hence lead to spermatogenesis damage. Our results were in line with previous related studies, which found that thoracic X-ray irradiation could destroy the structure of BTB and the expression of related proteins were significantly downregulated (Zhang et al., 2019a).

Inflammation is defensive pathological response and could happen when normal tissue subject injury (Candido and Hagemann, 2013). Abscopal effects of ionizing radiation may be initiated by cascade reactions of a series of inflammatory factors (Pouget et al., 2018). To investigate the mechanism of spermatogenesis damage induced by thoracic X-ray irradiation, we detect the levels of TNF-α, TGF-β1 and IL-10 in lung, serum and testis of mice. TNF-α is a pro-inflammatory cytokine and acts an important role in abscopal effects of radiation (Ansari et al., 2007). Song et al. found that after right thorax X-ray irradiation in mice, the release of TNF-α in the lungs induced the damage of the testis in a cascade reaction manner (Song et al., 2021). TGF-β1 is a multifunctional cellular inflammatory factor that can regulate cell proliferation and differentiation, which is also involved in ionizing radiation induced abscopal effects (Jiang et al., 2014). Zhang et al. found that after right thorax X-ray irradiation in rats, the concentration of TGF-β in serum significantly increased, and TM4 cells could be damaged when cultured with the serum (Zhang et al., 2019b). IL-10 is an important anti-inflammatory cytokine. We found that significant inflammatory reactions occurred in the lung, serum and testis of mice at 3 and 5 W after thoracic X-ray irradiation. Therefore, we speculate that the abscopal effects of thoracic X-ray irradiation, inflammatory factors produced in lungs can be released into the blood and then enter testicular tissues through the blood circulation, inducing a series of inflammatory responses.

It was reported that ionizing radiation induced abscopal effects were also associated with the level of oxidative stress (Yahyapour et al., 2018; Ping et al., 2020). MDA and T-SOD are important indicators of oxidative stress. It was reported the MDA content in lung was significantly changed after pelvic γ-ray irradiation (Ghobadi et al., 2017). Similarly, Abadi et al. (2018) found that pelvic irradiation could inhibit the activity of T-SOD and increase the level of MDA in cardiac and lung. Our study found that the levels of T-SOD in lung, serum and testis decreased, and the level of MDA increased significantly after thoracic X-ray irradiation, which suggested that oxidative stress may be involved in the process of spermatogenesis damage caused the abscopal effects of thoracic X-ray irradiation.

## 5 Conclusion

In conclusion, abscopal effects of thoracic X-ray irradiation on spermatogenesis in mice were obvious, which could significantly lower sperm quality, induce apoptosis of spermatogonial stem cells, damage testicular tissue, increase the permeability of BTB, and finally impair spermatogenesis, and a series of inflammation and oxidative stress factors were involved in the process.

## Data Availability

The original contributions presented in the study are included in the article/supplementary material, further inquiries can be directed to the corresponding author.
